# Circular RNAs in Sudden Cardiac Death Related Diseases: Novel Biomarker for Clinical and Forensic Diagnosis

**DOI:** 10.3390/molecules26041155

**Published:** 2021-02-21

**Authors:** Meihui Tian, Zhipeng Cao, Hao Pang

**Affiliations:** 1 Department of Forensic Genetics and Biology, School of Forensic Medicine, China Medical University, No.77 Puhe Road, Shenyang North New Area, Shenyang 110122, China; tianmh0619@163.com; 2 Department of Forensic Pathology, School of Forensic Medicine, China Medical University, No.77 Puhe Road, Shenyang North New Area, Shenyang 110122, China; zpcao@cmu.edu.cn

**Keywords:** circular RNA, biomarker, sudden cardiac death, diagnosis, forensic

## Abstract

The prevention and diagnosis of sudden cardiac death (SCD) are among the most important keystones and challenges in clinical and forensic practice. However, the diagnostic value of the current biomarkers remains unresolved issues. Therefore, novel diagnostic biomarkers are urgently required to identify patients with early-stage cardiovascular diseases (CVD), and to assist in the postmortem diagnosis of SCD cases without typical cardiac damage. An increasing number of studies show that circular RNAs (circRNAs) have stable expressions in myocardial tissue, and their time- and tissue-specific expression levels might reflect the pathophysiological status of the heart, which makes them potential CVD biomarkers. In this article, we briefly introduced the biogenesis and functional characteristics of circRNAs. Moreover, we described the roles of circRNAs in multiple SCD-related diseases, including coronary artery disease (CAD), myocardial ischemia or infarction, arrhythmia, cardiomyopathy, and myocarditis, and discussed the application prospects and challenges of circRNAs as a novel biomarker in the clinical and forensic diagnosis of SCD.

## 1. Introduction

Cardiovascular disease (CVD) is the most lethal noncommunicable disease, it globally being the greatest contributor to high morbidity and mortality [1,2,3]. Irregular cardiac activities restrict the blood supply, leading to sudden unexpected death caused by loss of cardiac function, known as sudden cardiac death (SCD) [4]. Globally, SCD accounts for 4–5 million deaths per year, and a considerable number of SCD events occur in asymptomatic individuals [1,3]. Clinically, due to the extremely rapid pathological process of SCD, most victims die within 1 h or even a few minutes of symptom onset, and survival rates remain low; thus, predicting and preventing the occurrence of SCD are the focus of clinical CVD [5,6,7,8]. In forensic practice, SCD identification is also one of the most important keystones and challenges, while autopsy can be completely negative in many cases, which needs further study [9]. Thus, the detection of novel biomarkers for SCD is urgently needed both in clinical and forensic medicine.

Many classical and novel biomarkers have emerged for predicting SCD, including protein, functional, and genetic biomarkers [1,10,11,12]. In 1976, Sanger and colleagues first introduced circular RNA (circRNA) in plant viroids and found that it has a special circular structure [13]. With the rapid advancements in biochemical methods, and the usage of high-throughput sequencing and microarray technologies, circRNAs are a research focus due to their unique structural and functional characteristics [14,15]. Since the 1990s, researchers have discovered more than 32,000 circRNAs from mammals, and their biological functions and mechanisms of action were gradually revealed [16,17,18,19,20,21]. The biological functions of circRNAs are continuously discovered and confirmed, mainly including the “miRNA sponge”, regulating transcription and gene expression, and regulating and encoding proteins [22,23,24,25]. Nowadays, the regulatory potential of circRNAs in the cardiovascular system is a hot topic and circRNAs, have emerged as novel players in diagnostic and therapeutic strategies for CVDs [26].

This review briefly introduces the biosynthesis and characteristics of circRNAs, and summarizes recent advances in the role of circRNAs in SCD-related diseases. Lastly, we focus on the potential of circRNAs as biomarkers in the clinical and forensic diagnosis of SCD, and discuss the current challenges, prospects, and directions for future research.

## 2. Introduction of CircRNA

**Figure 1:**
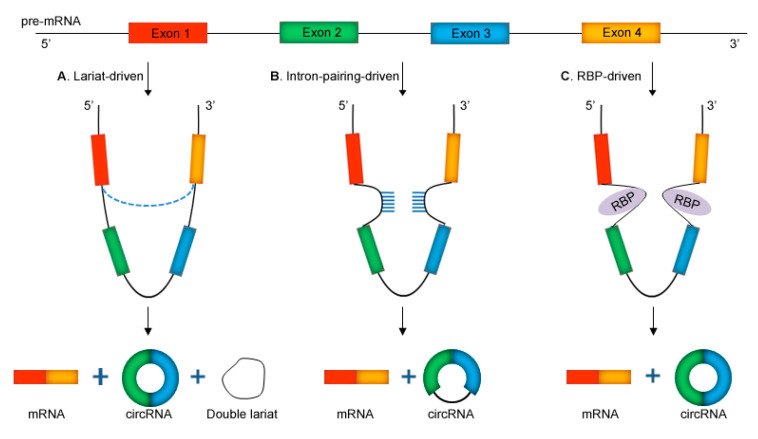
Biogenesis of circRNAs. (**A**) Lariat-driven model; (**B**) intron-pairing-driven model; (**C**) RNA-binding proteins (RBP)-driven model.

CircRNAs are a member of the non-coding RNAs (ncRNAs) family, formed by linear precursor message RNA (pre-mRNA) through reverse splicing [14]. Originally, circRNA was considered to be secondary byproducts of linear message RNA (mRNA). However further research has demonstrated that circRNA is an important product of pre-mRNA processing, and the processing mechanism of circRNA could compete with that of mRNA [27]. The covalently closed-loop structure of circRNAs, also known as backsplicing, is formed by the joining of the 3′ splice site to the 5′ splice site of mRNA [27,28]. Two models of circRNA biogenesis through backsplicing were first proposed by Jeck et al. in 2013, lariat-driven and intron-pairing-derived circularization (direct backsplicing), which sparked a new line of research into circRNA biogenesis [29]. The lariat precursor is also called exon skipping, which means that, during pre-mRNA synthesis, RNA is folded so that multiple exons are close to each other, forming a circular RNA intermediate, and then generate circRNA by lariat-like splicing (Figure 1A) [30]. In the direct backsplicing model, flanking intronic-sequence (such as the ALU sequence) complementarity leads to the formation of secondary structures in the pre-mRNA transcript, which primes the molecule for splicing via the appositional placing of two distant exons (Figure 1B) [28,29]. In addition, some scholars proposed relying on the cyclization mode of RNA-binding proteins (RBP), such as RNA polymerase II, the quaking protein, or the muscleblind protein, which can be combined with the RNA sequence, thereby promoting circRNA formation (Figure 1C) [27,31,32,33].

CircRNAs can be classified into three groups depending on their mode of biogenesis and their sequence: Exonic circular RNAs (ecircRNAs), intronic circular RNAs (ciRNAs), and exon–intron circular RNAs (EIciRNAs) [23,24,34,35]. EcircRNAs are only derived from exons in linear transcripts, and lack any introns in their sequences. The majority of circRNAs belong to this group and predominantly exist in the cytoplasm. In contrast, ciRNAs are not generated through backsplicing and lack exonic sequences. CiRNAs exist in the nucleus and have little enrichment for microRNA (miRNA) target sites. EIciRNAs contain both introns and exons in their sequences, which are mainly located in the nucleus. According to the circRNA location in the genome, circRNAs can also be grouped into intragenic and intergenic [36]. EcircRNAs, EIciRNAs, and ciRNAs are all intragenic circRNAs, which arise from sequences within the parental gene locus. CircRNAs derived from different genome intervals are collectively called intergenic circRNAs.

One of the most different features of circRNAs is the closed-loop structure, which is distinguished from that of other linear RNA molecules. Thus, circRNAs are insusceptible to gradation by RNA exonucleases and more stable compared to linear RNA molecules [19]. Researchers demonstrated that the average half-life of circRNAs can reach 50 h, which is a potential advantage of being biomarkers [37]. CircRNAs are conserved in mammals, and their expression exhibits tissue and disease specificity [20,38,39,40]. Sequencing results suggested that the most abundant circRNAs are expressed in the nervous system, heart, and tumor tissue [41,42,43]. The different expressions of circRNAs in myocardial tissue or body fluids by RNA sequencing or microarray analysis are summarized in Table 1. Werfel et al. found more than 9000 circRNAs in the hearts of rats, mice, and humans under different physiological and pathological conditions [20]. Results showed that about 30% circRNAs were conversed between mice and rats, and about 10% were conversed in all three species. In another study, researchers extracted 15,318 and 3017 specific circRNAs from 12 human heart-tissue samples and 25 mouse heart-tissue samples, respectively [44]. These results indicate that there is a large number of stable and highly expressed circRNAs in the human cardiovascular system, which suggests biomarker potential in CVDs. Furthermore, recent studies showed that circRNAs can be stably enriched in exosomes, and are widely present in body fluids such as blood, urine, saliva, and semen, which also lays a solid foundation for them being biomarkers [45].

## 3. CircRNA Serves as Biomarkers in SCD-Related Diseases

### 3.1. Coronary Artery Disease

The acute and persistent inadequate blood flow of coronary arteries may cause myocardial necrosis, which is known as myocardial infarction (MI). Worldwide, MI is the leading cause of mortality among all CVDs, and tends to occur in patients with coronary artery disease (CAD) [72]. With the development of high-throughput sequencing and chip technology, a large number of circRNAs related to CAD were discovered. Many circRNAs show strong involvement in the formation of atherosclerosis, such as circular antisense non-coding RNA (ANRIL) and circ_0003204 [73,74]. Yu and colleagues performed sequence analysis of circRNAs in the peripheral blood mononuclear cells of 70 CAD patients and 30 healthy controls, and identified 2283 downregulated and 85 upregulated circRNAs in total; many of the dysregulated circRNAs were involved in CAD pathophysiology [46]. Lin et al. divided CAD patients into control and test groups.On the basis of the results of routine inspection and the Gensini score, and found a total of 110 circRNAs to be differentially expressed in the two groups: 73 upregulated and 37 downregulated [47]. All these results indicated that circRNAs have critical roles in various biological CAD processes.

Research about circRNAs as potential biomarker for CAD is constantly developing (Table 2). A recent microarray study identified 3423 dysregulated circRNAs in five CAD patients and five matched healthy controls [48]. The expression levels of hsa_circ_0001946, hsa_circ_0008507 and hsa_circ_0000284 were significantly elevated in peripheral CAD blood leukocytes, consistent with their expressions in the peripheral blood, but exhibited significant differences in various populations. Zhao et al. analyzed circRNAs expression in peripheral blood of 12 CAD patients and 12 control individuals by RNA microarray, and found 22 differentially expressed circRNAs in CAD patients, including 12 upregulated and 10 downregulated circRNAs [49]. Among them, hsa_circ_0124644 expression was significantly upregulated in patients with CAD. The expression level was positively correlated with SYNTAX score and showed CAD diagnostic potential. Wang et al. found that a total of 624 circRNAs and 171 circRNAs were significantly upregulated and downregulated, respectively, in 24 CAD patients relative to 7 controls [50]. Hsa_circ_0001879 and hsa_circ_0004104 levels were significantly elevated, and the area under curve (AUC) was 0.703 and 0.700, respectively. Hsa_circ_ 0001879 level was significantly positively correlated with blood pressure, while hsa_circ_0004104 level was negatively correlated with blood HDL-C level, which suggested that hsa_circ_0001879 and hsa_circ_0004104 may be closely related to the pathological process of CAD. The combination of these two circRNAs with CAD risk factors could better discriminate CAD patients from healthy controls. In 200 patients with suspected stable CAD referred for coronary computed tomographic angiography exam, hsa_circ_0001445 showed remarkable stability in clinical specimens [75]. The plasma levels of the hsa_circ_0001445 are lower in patients with higher coronary atherosclerosis extension and severity, and performed as a biomarker of stable CAD. Zou et al. [76] found that compared with the control group, the expression level of circTCF25 (hsa_circ_0041103) in peripheral blood mononuclear cells of CAD patients was significantly reduced, and was negatively correlated with fasting blood glucose, creatine kinase and Gensini score. The sensitivity and specificity of circTCF25 as a CAD biomarker were 0.60 and 0.61, respectively. In addition, some researchers found that the expression of hsa_circRNA11783-2 was notably downregulated in the peripheral blood of patients with CAD and type 2 diabetes, predicting that it may serve as a “miRNA sponge” to adsorb miR-608 and miR-3907 to play a biological function [51,77].

### 3.2. Myocardial Infarction

In addition to coronary atherosclerosis, any other disease that can cause coronary-artery occlusion and subsequent loss of oxygen supply, such as coronary-artery vasospasm and anomalies can cause myocardial ischemia and even MI [79]. To identify circRNA expression changes in MI, scholars performed circRNA microarray analysis in sham and 3 days post-MI mouse hearts, and the expression of 1723 circRNAs was detected [52,80]. Of these, 82 circRNAs were consistently differentially expressed, including 41 upregulated circRNAs and 41 downregulated circRNAs. Among these, circFndc3b (mmu_circ_0001113) was significantly downregulated after validation by divergent primers and real-time RT-qPCR. The human circFNDC3B (hsa_circ_0001361) ortholog was also significantly decreased in the cardiac tissues of ischemic cardiomyopathy patients. Further findings indicated that the modulation of circFNDC3B expression may represent a potential strategy to promote cardiac function and remodeling after MI. In another study with 642 acute myocardial infarction (AMI) patients, Vasout et al. identified that the expression levels of myocardial infarction-associated circular RNA (MICRA, hsa_circ_0000615) were lower in MI patients compared with those of 86 healt hy volunteers [81]. Patients with low levels of MICRA were at high risk of left ventricle (LV) dysfunction, and MICRA level in the peripheral blood could predict LV dysfunction in 3–4 months after MI. Afterwards, they tested whether MICRA could risk-stratify MI patients. MICRA improved risk classification after MI, and provided an incremental value of this novel biomarker in future prognostication strategies [82].

The programed death of cardiomyocytes, including apoptosis and autophagy, critically contributes to the progressive loss of myocytes after the occlusion of a major epicardial coronary artery in MI [83]. Some circRNAs participate in the regulation of apoptosis, thereby regulating MI formation. For example, the expression of circACAP2 increased in cardiomyocytes after MI and promoted apoptosis by binding to miR-29 [84]. The circular RNA mmu_circ_0001878, also termed as Cdr1as, was derived from an antisense transcript of the cerebellar degeneration-related protein 1 gene at chromosome X (NC_000086.7) in mice [22,85]. Cdr1as was upregulated in MI mice with increased cardiac infarct size or cardiomyocytes under 6 h of hypoxia treatment [86]. Cdr1as overexpression could promote cell apoptosis and MI by mediating the regulation of miR-7a. Moreover, a recent study revealed that circ-Ttc3 (rno_circ_002317) was markedly upregulated in the ischemic myocardium of rats at 5 weeks post-MI, and in cardiomyocytes after being exposed to 6 h of ischemic insults [87]. The elevation of circ-Ttc3 expression protected cardiomyocytes against ischemia-related apoptotic death, while its downregulation aggravated cardiac dysfunction after MI, which seemed to be possible for the diagnostic and therapeutic targeting circ-Ttc3 in patients with ischemic heart disease (IHD). A recently discovered circRNA, circMACF1 (mmu_circ_0001258), was downregulated in the mouse myocardium at 3 days post-MI and in hypoxia-treated cardiomyocytes [88]. The overexpression of circMACF1 was effective in restoring cardiomyocyte apoptosis, and significantly reduced myocardial infarct size. Furthermore, Huang and colleagues found that circNfix (mmu_circ_0001704) level was decreased in the hearts of post-MI mouse, and its downregulation promoted cardiomyocyte proliferation and angiogenesis, and inhibited cardiomyocyte apoptosis after MI, which significantly attenuated cardiac dysfunction and improved prognosis [89]. Accordingly, circNfix might be a potent therapeutic target for restoring cardiac function and preventing heart failure after MI (Table 3).

### 3.3. Myocardial Ischemia

Though MI is the most frequent cause of IHD, an acute attack of myocardial ischemia without infarction can also lead to SCD [1]. However, studies about circRNAs involved in myocardial ischemia are rare, and most clinically referred to myocardial ischemia/reperfusion (MIR) injury (Table 4). In recent analysis of the circRNA expression profile in MIR rat models, a total of 10,387 circRNAs were identified, 69 circRNAs in the MIR group showed more than twofold differences compared with those in the sham group, and 28 circRNAs were detected to be differentially expressed in a protective group compared with those in the MIR group [53]. Among them, rno_circ_0000112, rno_circ_0005562, and rno_circ_0008515 were upregulated by MIR, but downregulated by protective group. Another study also detected that a total of 21 circRNAs were differentially expressed after 30 min ligation occlusion of the left anterior descending coronary artery, followed 4 h of reperfusion, namely, 14 upregulated circRNAs and 7 downregulated circRNAs [54]. Li et al. investigated the role of a circRNA transcribed from the sodium/calcium exchanger 1 (ncx1) gene, circNCX1 (mmu_circ_004295), with regard to heart function, cardiomyocyte apoptosis, and cardiac remodeling [90]. Results showed circNCX1 level was increased with the extension of the ligation time of the mouse heart and promoted cardiomyocyte apoptosis by acting as an endogenous miR-133a-3p sponge. Bai et al. confirmed that circular RNA DLGAP4 (circDLGAP4) levels were significantly decreased in the plasma of acute ischemic stroke patients (hsa_circ_0060180) and in a mouse stroke model (mmu_circ_0001098), and Wang et al. speculated that it might also act as a novel therapeutic target for MIR injury, which needs to be further proved by experimental evidence [91,92]. These findings suggest that circRNAs might contribute to MIR injury, and provide clues in circRNAs as novel biomarkers in myocardial ischemia diagnosis.

Autophagy is a well-established conserved mechanism that was demonstrated to have a critical role in many physiological and pathological processes of cardiovascular diseases. Zhou et al. analyzed circRNAs from ischemia/reperfusion (I/R)-and sham-treated mouse heart tissue by microarray, and reported that autophagy-related circular RNA (ACR, mmu_circ_006636) was markedly decreased after MIR injury [55]. ACR could also attenuate autophagy and cell death in cardiomyocytes, and reduce myocardial infarct size by targeting the Pink1-mediated phosphorylation of FAM65B. Another circular RNA associated with autophagy in MI is mmu_circ_101237, which is encoded by the cyclin dependent kinase 8 (CDK8) gene [56]. Mmu_circ_101237 level was significantly increased by anoxia/reoxygenation (A/R) injury in a time-dependent manner and mmu_circ_101237 mediated apoptosis in cardiomyocytes by activating autophagy.

Compared with the myocardial ischemia model caused by ligating coronary arteries, isoproterenol (ISO)-induced acute myocardial ischemia is reversible and relatively harmless, but can mimic the beginning stage of ischemia. Next-generation sequencing revealed that hsa_circ_0007623 was upregulated in hypoxia-induced human umbilical-vein endothelial cells and ISO-induced acute myocardial ischemia mice [93]. The expression of hsa_circ_0007623 promotes myocardial repair and improved cardiac function, and showed potential as a new biomarker for acute myocardial ischemia.

A large number of studies applied oxygen glucose deprivation (OGD) treatment to construct myocardial ischemia models in vitro, and circRNA expressions in OGD-treated cardiomyocytes could provide some ideas in seeking a safe and reliable method for the prevention and treatment of ischemia injury [94]. In a study on myocardial ischemia patients’ blood and OGD-treated rat H9c2 cells, the expression of circDENND2A (hsa_circ_0002142), which was highly expressed in HIF-1α induced glioma cells, was both significantly elevated in myocardial ischemia patients and conspicuously enhanced under OGD treatment. In another study, OGD treatment also induced the high expression of circZNF292 (rno_circ_009421) in the H9c2 cell line, and the overexpression of circZNF292 could release OGD-induced damage to the cardiomyocyte such as the inhibition of proliferation, apoptosis, and autophagy [95]. Moreover, hsa_circ_0010729 is a newly discovered circRNA that is associated with the anoxia-caused cell growth of vascular endothelial cells. Researchers also found that hsa_circ_0010729 expression was upregulated in OGD-treated human cardiomyocytes, and its silencing could protect cardiomyocytes from ischemic injury [96,97].

### 3.4. Arrhythmias

Given that fatal arrhythmias are responsible for most sudden deaths, identifying novel biomarkers for the occur-rence of arrhythmias is an important goal for the clinical diagnosis and forensic identification of SCD [98]. Though atrial fibrillation (AF) is the most common type of arrhythmia in the general population, ventricular arrhythmia (VA) is the major mechanism of SCD in IHD [57,98]. Some studies revealed that circRNAs participate in AF by the identification and characterization of circRNA expression profiles, and provide novel biomarkers and potential therapeutic targets for AF [99]. However, the expression and function of circRNAs in VAs were not experimentally confirmed.

By analyzing circRNA expression profiles in AF patients and healthy controls by circRNA microarray, Jiang and colleagues identified 537 upregulated circRNAs and 199 downregulated circRNAs in the AF patients, and many interacted with AF-related microRNAs [57]. Wu et al. collected atrial tissue from 7 AF patients and 7 matched controls, and identified a total of 280 circRNAs that were significantly differentially expressed by HiSeq/Proton RNA sequencing, including 46 upregulated and 234 downregulated, respectively [58]. After validation by real-time RT-qPCR on 35 pairs of AF patients and controls, two circRNAs, hsa_circ_24801 and hsa_circ_16247 showed significant dysregulation (Table 5). Expressed hsa_circ_0004113 and hsa_circ_0003965 levels were also consistent with Jiang’s results [57]. Costa et al. performed an unbiased study analyzing the expression profile for circRNAs in left-atrial biopsies from 8 patients with paroxysmal and permanent AF and 6 controls in sinus rhythm by RNA sequence [59]. Forty circRNAs were exclusively detected in permanent AF samples, which were generated by the noncanonical splicing of 18 coding genes. Among them, hsa_circ_012664 was also confirmed in Wu’s study [58]. In addition, scholars found 51 upregulated and 57 downregulated circRNAs by analyzing differential circRNA expression in atrial-tissue samples from patients with persistent AF with rheumatic heart disease and non-AF myocardium with normal hearts [60].

Zhang and colleagues investigated the differentially expressed profiles of circRNAs in the atrial appendage tissue of 9 AF patients with 6 non-AF patients, and identified an expression difference between the left and right atrial appendages [61]. Results identified 147 remarkably dysregulated circRNAs in total, including 102 upregulated and 45 downregulated circRNAs. Among them, hsa_circ_0005643 and novel_circ_0077334 were significantly increased, and might be involved in pathophysiological AF progress by binding with miR-221-5p. Genomewide profiling also revealed atrial fibrillation-related circular RNAs in both the left and right atrial appendages of AF patients and healthy controls with sinus rhythm [62]. There were 374 upregulated and 104 downregulated circRNAs in total in the left atrial appendages, and 267 upregulated and 268 downregulated circRNAs in the right atrial appendages. Notably, 20 upregulated and 3 downregulated circRNAs were identified to be differentially expressed in both the left and the right atrial appendage. Among them, hsa_circ_0000075 and hsa_circ_0082096 participated in AF pathogenesis via the TGF-beta signaling pathway, and hsa_circ_ 0003965 was associated with the glucagon signaling pathway.

In a recent study on the four left atrial appendages of patients with nonvalvular persistent atrial fibrillation (NPAF) and four normal controls, a total of 296 significantly dysregulated circRNA transcripts were obtained via RNA sequencing, 238 upregulated and 58 downregulated; hsa_circ_002085 and hsa_circ_001321 showed potential as NPAF biomarkers [63]. Furthermore, Khan et al. screened the plasma circRNA expression profiles of 769 patients with and without postoperative atrial fibrillation (PoAF), and found that the expression of hsa_circRNA_025016 was upregulated in patients with newly onset AF after isolated off-pump coronary-artery bypass grafting, and positively correlated to fasting blood glucose [64]. Results demonstrated that plasma hsa_circ_025016 holds potential as a biomarker for the prediction of PoAF.

### 3.5. Cardiomyopathy

Two-thirds of SCD patients have an abnormal heart structure, and cardiomyopathy is a major component [100,101]. Cardiomyopathy is defined as a myocardial disorder in which the heart muscle is structurally and functionally abnormal, in the absence of coronary-artery disease, hypertension, valvular disease, and congenital heart disease, sufficient to cause the observed myocardial abnormality [101]. It can be classified as hypertrophic cardiomyopathy (HCM), dilated cardiomyopathy (DCM), arrhythmogenic right ventricular cardiomyopathy (ARVC), restrictive cardiomyopathy (RCM), and nonclassifiable cardiomyopathy [101]. Mohsin et al. detected circRNAs in the left ventricles of 2 DCM, 2 HCM, and 2 control individuals by whole-transcriptome sequencing, and identified 826 circRNAs from 7130 putative ones; 43 out of 826 were differentially expressed in DCM compared with control samples, and 60 circRNAs in HCM compared with the control samples [33]. Nearly 10% of altered circRNAs were expressed from the titin gene, which undergoes highly complex alternative splicing. More interestingly, after validation by real-time RT-qPCR in a larger group of patients, a human panel of adult and fetal tissue samples, the authors confirmed the loss of circRNA formation from host genes CAMK2D (in DCM and HCM) and titin (mainly in DCM) in disease, and disease-regulated changes in circRNA production are independent of transcriptional regulation. Siede and colleagues also validated dynamically changed circRNAs in eight patients with DCM and eight nonfailing control hearts, while circRNAs from SLC8A1, CHD7, and ATXN10 increased relative to their host gene expression, while circDNA6JC decreases in a disease state [65]. However, limited to sample numbers, the potential of these circRNAs as biomarkers for DCM has not been experimentally proven. Sonnenschein et al. analyzed the expression levels of several circRNAs in serum samples from 64 patients with HCM and 53 healthy controls, and found circTMEM56, circDNAJC6, and circMBOAT2 to be significantly lower in patients with HCM than they were in healthy controls [78]. Moreover, after stratifying patients into a nonobstructive (HNCM) group and an obstructive form (HOCM) group, the levels of circTMEM56 and circDNAJC6 circRNAs were negatively correlated with the severity of left ventricular obstruction and thickness of interventricular septum in the HOCM group, and could serve as indicators of disease severity in patients with HOCM. Results indicated good performance of these three circRNAs as HCM biomarkers, which may facilitate the clinical diagnosis of HCM.

In a mouse model of doxorubicin-induced cardiomyopathy (DOXIC), Zeng et al. found that circ-Amotl1 (hsa_circ_0004214) decreased in the cardiac tissue of mice after treating doxorubicin (Dox) for 14 days [66]. Circ-Amotl1 expression was also protective against apoptosis, the formation of collagen, and LV dysfunction. In a recent study on DOXIC, circular RNA sequencing was performed to profile circRNA expression in the hearts of cancer patients suffering from DOXIC and healthy donors. In total, 356 circRNAs were differentially expressed, namely, 207 upregulated and 149 downregulated [67]. Among the identified circRNAs, circITCH (hsa_circ_0001141) was consistently abundant in human primary cardiomyocytes, human left ventricle tissue, and human-induced pluripotent stem-cell-derived cardiomyocytes (hiPSC-CMs). CircITCH level was lower in the hearts of cancer patients suffering from DOXIC than that in the hearts of DCM, HCM, and healthy donors. These findings also unveiled that circ-Amotl1 and circITCH might be novel diagnostic and therapeutic molecular targets that are amenable to DOXIC.

Diabetic cardiomyopathy is characterized by myocardial fibrosis, ventricular remodeling, and cardiac dysfunction, which is in the absence of hypertensive-heart, coronary-artery, and valvular-heart diseases [102]. The development of cardiac fibrosis is the key event in the pathogenesis of diabetic cardiomyopathy. Yang et al. identified that hsa_circ_0076631 levels increase in both high-glucose-treated cardiomyocytes and the serum of diabetic patients, and might facilitate precise gene diagnosis and therapeutic targets for diabetic car-diomyopathy [103]. Zhou et al. utilized circRNAs microarray analysis to detect the circRNA expression profile in a di-abetic mouse myocardium, and found that a total of 43 circRNAs were differentially expressed, including 24 upregulated circRNAs and 19 downregulated circRNAs, compared with the control groups [68]. Among them, mmu_circ_010567 was evidently upregulated and involved in the pathological process of myocardial fibrosis through negatively regulating miR-141 by targeting TGF-β1. Another circRNA profiling array performed on a diabetic cardi-omyopathy mouse myocardium revealed 45 increased circRNAs and 31 decreased circRNAs [69]. Real-time RT-qPCR verified that mmu_circ_000203 was dramatically upregulated in this disease state, and enhanced the expressions of Col1a2, Col3a1, and α-SMA in cardiac fibroblasts by acting as sponge to miR-26b-5p. Mechanistically, Li et al. also found that mmu_circ_000203 could exacerbate cardiac hypertrophy by suppressing miR-26b-5p and miR-140-3p binding to Gata4 [104]. These results suggested that mmu_circ_000203 might be a potential target for the prevention and treatment of cardiac fibrosis in diabetic cardiomyopathy.

Furthermore, of 643 circRNAs, scholars identified 114 upregulated and 151 downregulated that were expressed in the left ventricle myocardium of a mouse with alcoholic cardiomyopathy (ACM) [70]. These tremendous changes of circRNA expression in the heart further indicated that circRNAs might play significant biological roles in the development of ACM, but biomarker potential in ACM needs further study (Table 6).

### 3.6. Myocarditis

Myocarditis represents myocardial inflammation caused by diverse etiologies, and it is a major cause of SCD in children and young adults [106]. Zhang et al. analyzed the expression patterns of circRNAs in leukocytes separated from the peripheral blood samples of children with fulminant myocarditis and healthy volunteers by circRNA micro-array, and found that 3173 circRNAs showed differential expression, namely, 2281 upregulated and 892 downregulated circRNAs [71]. Among seven selected circRNAs, hsa_circ_0071542 was confirmed to be upregulated in the fulminant myocarditis group versus in control patients, while hsa_circ_0014350 was the contrary. In addition, hsa_circ_0071542 expression might be associated with the severity of myocardial damage, and serve as a biomarker of fulminant myocarditis.

Shi et al. also found that circANKRD36 expression was prominently raised in lipopolysaccharide (LPS) pre-treated H9c2 cells, and silencing circANKRD36 alleviated LPS-induced apoptosis and inflammatory injury by inhibiting the p38MAPK/NF-κB pathway via up-regulating miR-138 [105]. These findings uncovered a novel molecular mechanism of circANKRD36 and miR-138 effect on myocarditis, and provided an innovative target for clinical treatment.

## 4. CircRNAs as Novel Biomarkers in Postmortem SCD Diagnosis

### 4.1. CircRNA Applications in Forensic Medicine

Due to the special molecular structure of circRNAs, forensic scientists pay attention to their study and seek to apply them to solving problems related to forensic identification. In forensic pathology, the analysis of the cause of death and the prediction of postmortem interval (PMI) are major foci, while individual identification and paternity tests are crucial for forensic genetics. Tu et al. evaluated the stability of multi-RNA markers in the heart, liver, and skeletal-muscle tissue of mice within 8 days after death, and concluded that miRNAs and circRNAs were more stable as reference genes than as other kinds of RNAs regarding PMI estimation [107]. They also established mathematical models of PMI estimation using the above selected reference genes and target biomarkers, which could increase PMI accuracy in advanced stages [108]. CircRNAs could also be used in the identification of forensically relevant tissue and body fluids on the basis of their high abundance, remarkable stability, and tissue-specific expression profiles [109]. In order to find tissue-specific circRNAs molecular markers, Gao et al. investigated 25 human tissue-specific circRNA expressions and tested circRNA stability. A total of 8 kinds of circRNAs were successfully verified in the heart, brain, liver, skin, and skeletal muscles.

The experiment results suggested that circRNAs had the ideal stability and different expression levels in human organs and tissue types, and could be used as new genetic markers for forensic humoral identification [110]. Zhang et al. investigated whether the inclusion of circRNAs in mRNA profiling improved the detection of biomarkers, including δ-aminolevulinate synthase 2 (ALAS2) and matrix metallopeptidase 7 (MMP7), in body-fluid identification. Results showed that the inclusion of circRNAs in mRNA profiling could facilitate the detection of mRNA markers in forensic body-fluid identification by using serial dilutions, mixed samples, menstrual bloodstains, and degraded and aged samples [111]. Further studies demonstrated that the expression of circRNAs could help in identifying common biofluids, even though all linear transcripts were completely erased by RNase R treatment [109,112]. In a recent study, researchers performed RNA sequence analysis on 13 biologically independent human peripheral whole blood specimens, and 7 age-correlated circRNA markers were identified. These results indicated that circRNAs could be used as a candidate marker for age prediction in peripheral blood, which provides theoretical basis for screening new molecular markers for age prediction in forensic medicine [113].

### 4.2. Current Biomarkers in Postmortem SCD Diagnosis

Various candidate biomarkers for SCD were discovered and applied in CVD diagnosis and treatment in clinical cardiology. However, there are relatively few globally recognized biomarkers for forensic postmortem SCD identification, though some were reported to improve diagnostic sensitivity, accuracy, and validity in forensic medicine. Biochemical markers such as cardiac troponin I (cTnI), creatine kinase MB (CK-MB), and N-terminal pro-B-type natriuretic peptide (NT-proBNP) are the most commonly used in postmortem SCD diagnosis in forensic practice [11,114,115]. The levels of these indicators in the blood or pericardial fluid are an important auxiliary for the diagnosis of SCD. Another kind of classical biomarker is inflammatory biomarker, such as C-reactive protein, high-sensitive C-reactive protein, and interleukin 6 [109]. Inflammation plays a pivotal role in the pathophysiology of most kinds of CVDs. Therefore, it is easy to speculate a link between inflammatory markers and SCD.

In the past decade, techniques of molecular biology also improved forensic medicine, especially in the determination of the cause of death [116]. Molecular-tissue changes in pathophysiology by immunohistochemical detection gradually participate in the postmortem identification of SCD. Several immunohistochemical markers, such as growth-associated protein-43 (GAF-43), connexin 43 (CX43), vascular endothelial growth factor (VEGF), and hypoxia-inducible factor 1-alpha (HIF-1α) were proposed for improving the postmortem detection of myocardial-ischemia-related SCD [117]. Genetic and epigenetic markers are also gaining attention. On the one hand, genomewide studies detected the association of many single-nucleotide polymorphisms with an increased risk of SCD [118,119,120,121]. On the other hand, epigenetic mechanisms, including DNA methylation, histone modification, and chromatin remodeling were also found to be crucial for the regulation of gene expression in SCD [122]. Some nc-RNAs were also reported to present high accuracy in discriminating SCD from AMI and healthy control [123].

### 4.3. Opportunities and Challenges

Studies on circRNAs as postmortem diagnostic biomarkers of SCD are lacking and face immense challenges, despite their tremendous potential as clinical CVD markers. An ideal biomarker needs to have high sensitivity and specificity, and should not be affected by many factors such as patient gender, age, lifestyle, and genetic background [124]. It would be even better if a biomarker could reflect the severity of the disease stage or even predict the outcome [26]. Furthermore, safety and noninvasiveness are the basic conditions for their wide clinical application. In this context, circRNAs are suitable biomarkers in CVD diagnosis. First, the presence of thousands of circRNAs was described in CVD with tissue- and time-specific expressions, and many of them showed high sensitivity and specificity. Second, due to the closed circular molecule, circRNAs are resistant to RNA exonucleases, and hence have better stability than that of other types of ncRNAs [19]. Additionally, circRNAs are enriched in exosomes, which can be collected by several biological fluids such as blood, urine, saliva, breast milk, and semen [45]. Detecting the circulating circRNAs expression in body fluids not only facilitates the clinical diagnosis, but also provides the possibility for the detection of special forensic biological materials (such as pericardial and vitreous body fluids). Moreover, because of the competition with mRNA in the processing mechanism, tissue and body-fluid samples with few mRNA expression changes may have significant alteration of corresponding circRNA levels, which excavates new biomarkers [24,109]. However, more research on the exploration of circRNAs as potential biomarkers should be carried out on large amounts of demographic data. First, although the expression level of circular RNAs undergoes a great change during the occurrence of various cardiovascular diseases, patients’ physiological conditions need to be more explicit, including criteria such as age, gender, diet habits, and genetic background. Second, standard operating procedures and guidelines for best practices, in addition to automated and standardized assays, are also needed, including the selection, collection, and treatment of samples, test methods on circRNA identification and quantification, and normalization for endogenous or external references. Third, the establishment of a reliable reference-value range can ultimately ensure the wide application of circRNAs as biomarkers in SCD.

One of the most important of these challenges is that samples in forensic medicine are often affected by postmortem changes; thus, some widely used biomarkers may have specific limitations due to hemolysis or degradation or be impaired by a wide variety of noncardiac causes [125,126,127]. Although the average half-life of a circular RNA can reach 50 h, many factors, such as the length of the amplified products, primer specificity, and endogenous controls, can affect the actual detection level [37,107,127]. Good postmortem stability is important to determine whether a clinically validated circRNA can be used in forensic analysis. A previous study evaluated the stability of several circRNAs, including circ-AFF1, in the hearts of mice within 8 days after death, and concluded that, as reference genes, circRNAs were more stable than other kinds of RNAs are in dead bodies [107,108]. Studies also reported that circRNAs presented relatively consistent and stable expression profiles in formalin-fixed paraffin-embedded tissues compared with their corresponding linear transcripts [128]. However, real-world conditions are much more complicated than laboratory conditions, especially with postmortem cases. Using short amplicons and standardized RT-qPCR assays in autopsy cases might improve the possibility of performing accurate quantitative analysis of circRNAs [128]. On the plus side, forensic scientists can obtain sufficient tissue samples through autopsies, even including some unconventional samples such as pericardial fluid, which also expands the conditions for the selection of appropriate biomarkers. High throughput methods such as RNA sequencing and microarray analysis for the expression profile of circRNAs in cardiac tissue of SCD victims may be extremely valuable. The thorough macro- and histo-pathological postmortem investigations could also provide more clues for the application of circRNAs as novel biomarkers. So far, there is no research on circRNA expression in the pericardial fluid, which may be another direction of circRNAs as potential biomarkers in the postmortem diagnosis of SCD.

In conclusion, increasing evidence indicates that circRNAs emerge as novel biomarkers in clinical diagnosis of CVDs, and have the potential of being biomarkers for postmortem diagnosis of SCD in forensic medicine, even though several questions remain unanswered. Further studies are required to be carried out with this specific research purpose.

## Figures and Tables

**Table 1:** Different expressions of circRNAs in sudden cardiac death (SCD) related diseases by RNA sequencing or microarray analysis.

Diseases	Species	Samples	Sample Capacity	Technique	Expression Regulation			Representative CircRNAs	Reference
			Case/Control		DE	UR	DR		
CAD	human	PBMC	70/30	sequence	2368	2283	85	hsa_circ_0003915, hsa_circ_001272, hsa_circ_0103771, hsa_circ_0006251, hsa_circ_0066529, hsa_circ_0030042	[46]
CAD	human	PB	20/20	microarray	110	73	37	hsa_circ_0030769, hsa_circ_0079828, hsa_circ_15486-161, hsa_circ_0122274, hsa_circ_16316-13, hsa_circ_0140538	[47]
CAD	human	PB	5/5	microarray	3423	-	-	hsa_circ_0125589, hsa_circ_0001946, hsa_circ_0008507	[48]
CAD	human	PB	12/12	microarray	22	12	10	hsa_circ_0082081, hsa_circ_0113854, hsa_circ_0124644, hsa_circ_0098964, hsa-circRNA5974-1	[49]
CAD	human	PBMC	24/7	microarray	795	624	171	hsa_circ_0001879, hsa_circ_0004104, hsa_circ_004432	[50]
CAD	human	PB	6/6	microarray	40	13	27	hsa_circRNA11783-2, hsa_circRNA6510-1	[51]
MI	mouse	Myo	2/2	microarray	82	41	41	mmu_circ_0001113	[52]
MIR	rat	Myo	2/2	sequencing	69	36	33	rno_circ_0000112, rno_circ_0005562, rno_circ_0008515	[53]
MIR	mouse	Myo	3/3	sequencing	21	14	7	novel_circ_0020886, novel_circ_0008516, novel_circ_0002498	[54]
MIR	mouse	Myo	1/1	microarray	-	-	-	mmu_circ_006636	[55]
MIR	mouse	PCM	1/1	microarray	-	-	-	mmu_circ_400095, mmu_circ_101512, mmu_circ_100714, mmu_circ_405755, mmu_circ_101237	[56]
AF	human	Myo	3/3	microarray	736	537	199	hsa_circ_100612, hsa_circ_405917	[57]
AF	human	Atr	7/7	sequencing	280	46	234	hsa_circ_24801, hsa_circ_16247	[58]
AF	human	left-Atr	8/6	sequencing	40	-	-	hsa_circ_0025470, hsa_circ_0035132, hsa_circ_0035148, hsa_circ_0057344, hsa_circ_0112651, hsa_circ_0112664	[59]
AF	human	Atr	9/6	sequencing	108	51	57	hsa_circ_20118, hsa_circ_17558, hsa_circ_16688 ,hsa_circ_11109, hsa_circ_11017, hsa_circ_11058	[60]
AF	human	Atr	9/6	sequencing	147	102	45	hsa_circ_0005643, novcl_circ_0026284, novel_circ_0077334, novel_circ_0046852, novel_circ_0068696, novel _circ_0055884	[61]
AF	human	Atr	10/10	sequencing	478 (left)	374	104	hsa_circ_0004270, hsa_circ_0000075, hsa_circ_0030254, hsa_circ_0007271, chr6:129371063-129419560	[62]
					535 (right)	267	268		
					23 (both)	20	3		
AF	human	Atr	4/4	sequencing	296	238	58	hsa_circ_002085, hsa_circ_001321	[63]
AF	human	blood	15/15	microarray	31	24	7	hsa_circ_025016, hsa_circ_404686, hsa_circR_000367, hsa_circ_001729, hsa_circ_100790, hsa_circ_030162, hsa_circ_100789, hsa_circ_104270, hsa_circ_102049	[64]
HCM	human	LV	2/2	sequencing	60	34	26	hsa_circ_0003101, chr9:108484902-108467878	[33]
DCM	human	LV	2/2	sequencing	43	15	28	chr4:121675708-121732604, chr2:179542852-179586861	[33]
DCM	human	hiPSC-CMs	26 ^1^	sequencing	-	-	-	circNCX1, circCHD7, circATXN10, circDNAJ6C	[65]
DOXIC	human	Myo	10/17 ^2^	microarray	-	-	-	hsa_circ_0004214	[66]
DOXIC	human	Myo	3/3	sequencing	356	207	149	hsa_circ_0001141	[67]
diabetic CM	mouse	Myo	4/4	microarray	43	24	19	mmu_circ_010567	[68]
diabetic CM	mouse	Myo	4/4	microarray	76	45	31	mmu_circ_000203	[69]
ACM	mouse	Myo	3/3	microarray	265	114	151	mmu_circ_011978, mmu_circ_011979, mmu_circ_011977, mmu_circ_011982, mmu_circ_011976, mmu_circ_011975,	[70]
fulminant myocarditis	human	PB	3/3	microarray	3172	2281	892	hsa_circ_0071542, hsa_circ_0014350, hsa_circ_0073029	[71]

^1^ Differentiation time course (0–17 day), 3 replicates each; ^2^ neonatal/mature postnatal human. Abbreviation: DE, different expression; UR, up-regulated; DR, down-regulated; CAD, coronary artery disease; MI, myocardial infarction; MIR, myocardial ischemia/reperfusion; HCM, hypertrophic cardiomyopathy; DCM, dilated cardiomyopathy; DOXIC, doxorubicin-induced cardiomyopathy; CM, cardiomyopathy ACM, alcoholic cardiomyopathy; PBMC, peripheral blood mononuclear cell; PB, peripheral blood; PBL, peripheral blood; Myo, myocardial tissues; LV, left ventricle; Atr, atrial tissues; PCM, primary cardiomyocyte; hiPSC-CMs, human induced pluripotent stem cell derived cardiomyocytes.

**Table 2:** The diagnosis value of circRNAs in SCD related diseases.

Diseases	CircRNA	Samples	Sample Capacity	Expression Regulation	AUC (95% CI)	Sensitivity	Specificity	*p*-Value	Reference
			Case/Control						
CAD	hsa_circ_0001946	PBL	100/100	up	0.71 (0.64–0.79)	0.85	0.52	-	[48]
CAD	hsa_circ_0000284	PBL	100/100	up	0.68 (0.61–0.76)	0.66	0.71	-	[48]
CAD	hsa_circ_0008507	PBL	100/100	up	0.75 (0.68–0.82)	0.86	0.60	-	[48]
CAD	hsa_circ_0124644	PB	115/137	up	0.872 (0.785–0.960)	0.867	0.767	<0.001	[49]
CAD	hsa_circ_0082081	PB	115/137	up	0.66 (0.522–0.798)	0.833	0.433	0.033	[49]
CAD	hsa_circ_0113854	PB	115/137	up	0.689 (0.555–0.823)	0.867	0.50	0.012	[49]
CAD	hsa_circ_0098964	PB	115/137	up	0.82 (0.707–0.933)	0.80	0.867	<0.001	[49]
CAD	hsa_circRNA5974-1	PB	115/137	up	0.743 (0.619–0.867)	0.633	0.733	0.001	[49]
CAD	hsa_circ_0001879	PBMC	412/290	up	0.703 (0.656–0.750)	0.831	0.543	<0.001	[50]
CAD	hsa_circ_0004104	PBMC	412/290	up	0.700 (90.646–0.755)	0.707	0.614	<0.001	[50]
CAD	hsa_circRNA11783-2	PBMC	60/81	down	-	-	-	-	[51]
CAD	hsa_circ_0001445	PB	74/126^1^	down	0.589 (0.506–0.671)	-	-	< 0.001	[75]
CAD	hsa_circ_0041103	PBMC	342/246	down	0.62 (0.57–0.67)	0.60	0.61	<0.001	[76]
AF	hsa_circ_025016	plasma	75/295	up	0.802 (0.798–0.806)	0.794	0.776	<0.001	[64]
HCM	circDNAJC6	serum	64/53	down	0.819 (0.725–0.912)	-	-	<0.001	[78]
HCM	circTMEM56	serum	64/53	down	0.738 (0.621–0.856)	-	-	<0.001	[78]
HCM	circMBOAT2	serum	64/53	down	0.756 (0.653–0.860)	-	-	<0.001	[78]

^1^ Stratified by segment stenosis score (SSS), SSS > 5 vs. SSS ≤ 5. Abbreviation: CAD, coronary artery disease; AF, atrial fibrillation; HCM, hypertrophic cardiomyopathy; PBMC, peripheral blood mononuclear cell; PB, peripheral blood; PBL, peripheral blood.

**Table 3:** CircRNAs as potential biomarkers in myocardial infarction validated by real-time RT-qPCR.

CircRNAs	Samples	Sample Capacity	Expression	Functions	Reference
		Case/Control	Regulation		
mmu_circ_0001113	3 d post-MI mice heart	5/8	down	induces cardiomyocyte apoptosis and reduce neovascularization	[52]
hsa_circ_0000615	MI patient whole blood	327/86	down	predicts of LV dysfunction, improves risk classification after MI	[81] [82]
circACAP2	MI rat heart	-/-	up	promotes apoptosis	[84]
mmu_circ_0001878	24 h post MI mice heart	10/10	up	overexpression in vivo increased cardiac infarct size	[86]
rno_circ_002317	5 w post-MI rats heart	8/6	up	restrains ischemic cardiac injury	[87]
mmu_circ_0001258	3 d post-MI mice heart	6/6	down	impairs the progression of AMI by modulating the miR-500b-5p/EMP1 axis	[88]
mmu_circ_0001704	7 d post-MI mice heart	6/6	down	downregulation of it promotes cardiomyocyte proliferation and angiogenesis, and inhibits cardiomyocyte apoptosis after MI	[89]

Abbreviation: MI, myocardial infarction.

**Table 4:** CircRNAs as potential biomarkers in myocardial ischemia models validated by real-time RT-qPCR.

Disease Models	CircRNAs	Samples	Sample Capacity	Expression Regulation	Functions	Reference
			Case/Control			
myocardial ischemia	mmu_circ_004295	30 min ischemia of mouse heart	6/6	up	promotes cardiomyocyte apoptosis	[90]
myocardial ischemia	hsa_circ_0007623	ISO intraperitoneally injected of mouse heart	6/6	up	promotes myocardial repair and improves cardiac function	[93]
MIR	mmu_circ_006636	60 min ischemia follow 3 h/1 h reperfusion mice heart	6/6	down	protects the heart from ischemia/reperfusion injury and reduces myocardial infarct sizes	[55]
MIR	mmu_circ_101237	primary cardiomyocyte of mice	3/3	up	attenuates autophagy and cell death in cardiomyocytes, and reduces myocardial infarct size	[56]
MIR	hsa_circ_0060180	acute ischemic stroke patients plasma	26/26	down	participates in apoptosis	[91]
OGD	hsa_circ_0002142	acute myocardial ischemia human blood	25/25	up	enhances OGD-induced cell viability and migration, but declines OGD-induced apoptosis	[94]
OGD	rno_circ_009421	rats H9c2 cells	3/3	up	releases OGD-induced damage and down-regulates apoptosis and autophagy	[95]
OGD	hsa_circ_0010729	human cardiomyocytes	3/3	up	silence of it could help protect cardiomyocytes from ischemic injury	[96]

Abbreviation: MIR, myocardial ischemia/reperfusion; OGD, oxygen glucose deprivation; ISO, isoproterenol.

**Table 5:** Different expressions of circRNAs in atrial fibrillation validated by real-time RT-qPCR.

CircRNAs	Samples	Sample Capacity	Expression	Reference
		Case/Control	Regulation	
hsa_circ_100612, hsa_circ_405917	human left atrial appendages	3/3	down	[57]
hsa_circ_008132, hsa_circ_104052, hsa_circ_101021, hsa_circ_101020, hsa_circ_102341, hsa_circ_404747, hsa_circ_002641, hsa_circ_079477	human left atrial appendages	3/3	up	[57]
hsa_circ_16247, hsa_circ_24801	human atrial tissues	35/35	up	[58]
hsa_circ_0005643, novcl_circ_0026284, novel_circ_0077334, novel_circ_0068696, novel_circ_0055884	human atrial tissues	15/15	up	[61]

**Table 6:** CircRNAs as potential biomarkers in cardiomyopathies and myocarditis validated by real-time RT-qPCR.

Diseases	CircRNA	Samples	Sample Capacity	Expression Regulation	Functions	Reference
			Case/Control			
ICM	hsa_circ_0006156	human heart tissues	7/4	down	reduces left ventricular functions	[52]
DCM	circNCX1 ratio ^1^, circCHD7 ratio, circATXN10 ratio	human heart tissues	8/8	up	-	[65]
DCM	circDNAJ6C ratio	human heart tissues	8/8	down	-	[65]
DOXIC	hsa_circ_0004214	mouse heart tissues	10/10	down	educes the enlarged left ventricle, and facilitates nuclear translocation of AKT and PDK1	[66]
DOXIC	hsa_circ_0001141	human heart tissues	3/3	up	ameliorates DOX-induced cardiomyocyte injury and dysfunction	[67]
Diabetic CM	hsa_circ_0076631	diabetic patient serum	10/10	up	mediates pyroptosis of diabetic cardiomyopathy by functioning as a competing endogenous RNA	[103]
Diabetic CM	mmu_circ_010567	mouse heart tissues	10/10	up	participates in the pathogenesis of myocardial fibrosis	[68]
Diabetic CM	mmu_circ_000203	mouse heart tissues	8/8	up	enhances the expression of fibrosis-associated genes by depressing targets of miR-26b-5p, Col1a2 and CTGF, in cardiac fibroblasts	[69]
fulminant myocarditis	hsa_circ_0071542	human heart tissues	8/8	up	may be associated with the severity of fulminant myocarditis	[71]
myocarditis	circANKRD36	rats H9c2 cells	3/3	up	silencing it alleviates apoptosis and inflammatory injury induced by lipopolysaccharide	[105]

^1^ Ratio refers to circRNA/(circRNA+LinearRNA). Abbreviation: ICM, ischemic cardiomyopathy; DCM, dilated cardiomyopathy; DOXIC, doxorubicin-induced cardiomyopathy; DOX, doxorubicin; CM, cardiomyopathy.

## Data Availability

Not applicable.
